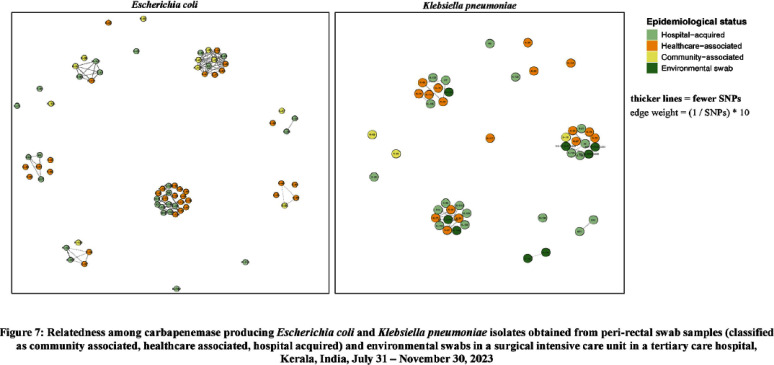# CRE colonization on admission and acquisition among surgical intensive care unit patients in an Indian tertiary care hospital

**DOI:** 10.1017/ash.2025.191

**Published:** 2025-09-24

**Authors:** Fabia Edathadathil, Lindsey Hall, Emily Benedict, Jobin Jacob, Devendhu Suresh, Yathu Krishna, Jacaranda Van Rheenen, Ige George, Jennie H. Kwon, Margaret Olsen, Anil Kumar, Surbhi Leekha, Gautam Dantas, Veeraraghavan Balaji, Sudheer Vayoth, Sanjeev Singh, David Warren, Sumanth Gandra

**Affiliations:** 1 Washington University in St. Louis; 2 AIMS, Kochi, India; 3 Washington University - School of Medicine; 4 University of Maryland Baltimore; 5 CMC Vellore; 6 Amrita Institute of Medical Sciences; 7 Washington University School of Medicine in St. Louis

## Abstract

**Introduction:** Studies examining carbapenemase producing carbapenem resistant Enterobacterales (CP-CRE) transmission incorporating clinical and genomic data in Indian hospitals are lacking. We investigated the prevalence, risk factors for CP-CRE peri-rectal colonization on admission and acquisition during hospital stay and genomic epidemiology of CP-CRE isolates in an adult surgical intensive care unit (SICU) in a tertiary-care hospital in India. **Methods:** SICU patients admitted from July 31 to November 30, 2023 were prospectively enrolled. Peri-rectal swabs (PRS) were collected at SICU admission and discharge, and hospital discharge. Environmental sampling of sinks was performed. Swabs were plated on selective agar (CHROMagarTMmSuperCARBATM) for CP-CRE isolation. Whole genome sequencing of CP-CRE isolates was performed to investigate antimicrobial resistance gene (ARG) abundance, strain typing (ST), and relatedness classified by community-associated (CA), healthcare-associated (HCA), hospital-acquired (HA), and environmental isolates. **Results:** 56 (28%) of 203 enrolled patients were colonized with CP-CRE on SICU admission. Among 147 admission-negative patients, 113 had repeat PRS testing > = 1 times during their stay; 43 (29%; 43/147) acquired CP-CRE (Figure 1). The predominant organism in admission and acquisition cases was Escherichia coli (52%) and Klebsiella pneumoniae (37%), respectively (Figure 2). Previous hospitalization = 2 antibiotics (aOR 2.77; 95%CI 1.12-6.82) were associated with admission CP-CRE colonization (Figure 3). In Cox regression analysis hospital stay before SICU admission was associated with CP-CRE acquisition in the SICU, but no risk factor was associated with acquisition during the entire hospital stay (Figure 4). Abundance of ARGs was lower in CA CP-CRE isolates compared to HCA, HA and environmental isolates (Figure 5). blaNDM and blaOXA genes were present in 79% (99/126) and 29% (36/126) of isolates, respectively; blaNDM-5 was the most common carbapenemase [65 (52%) of 126 isolates] (Figure 6A). E. coli ST410, which was associated with HA and HCA classifications was the most frequent ST (n=17) and 70% (12/17) carried NDM (Figure 6B). Twenty-seven E. coli and 17 K. pneumoniae isolates were separated by 20 or fewer core genome single-nucleotide polymorphisms, indicating potential relatedness amongst CP-CRE (Figure 7). **Conclusion:** More than 25% of SICU patients were colonized with CP-CRE on admission and also acquired CP-CRE during hospital stay. Healthcare-related CP-CRE isolates carried more resistances genes with NDM being the most commonly detected resistance gene in this cohort. Small sample size limited our understanding of risk factors associated with CP-CRE acquisition in hospital.

**Figure:**
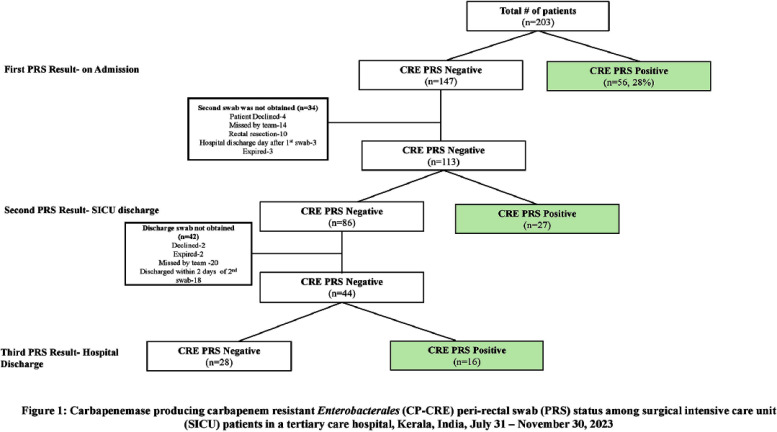

**Figure:**
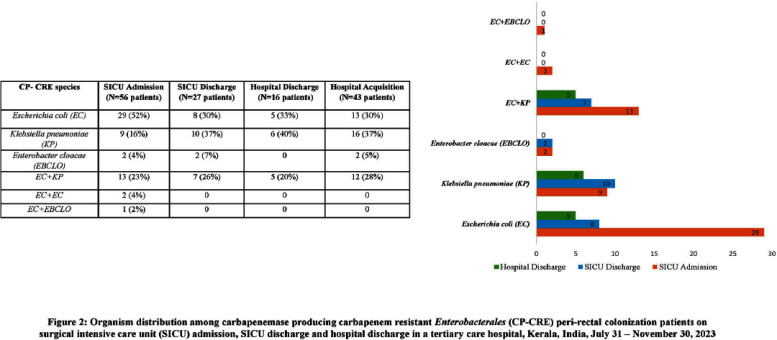

**Figure:**
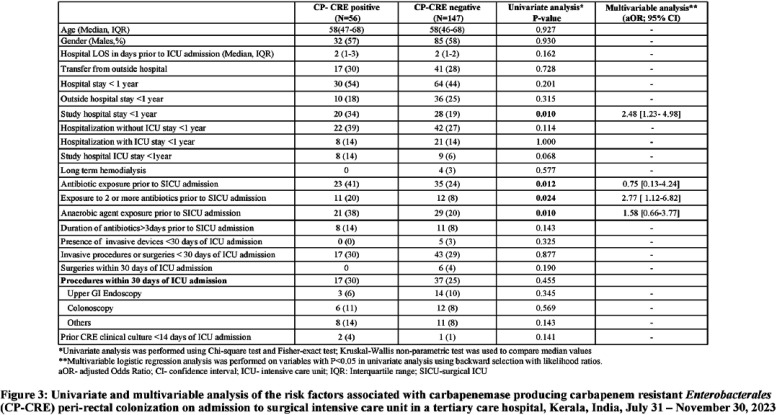

**Figure:**
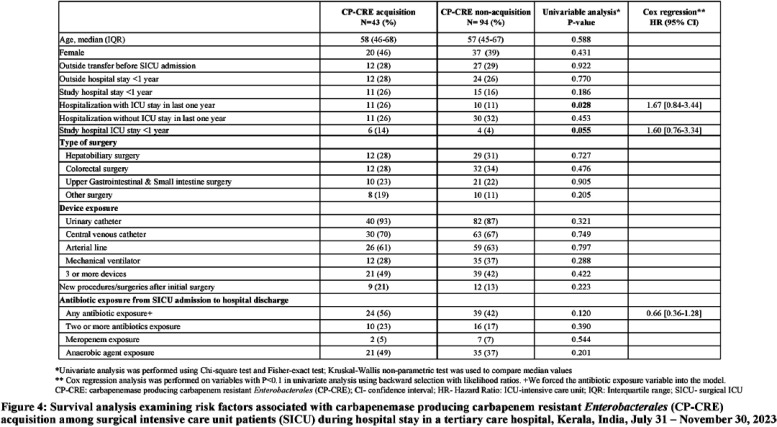

**Figure:**
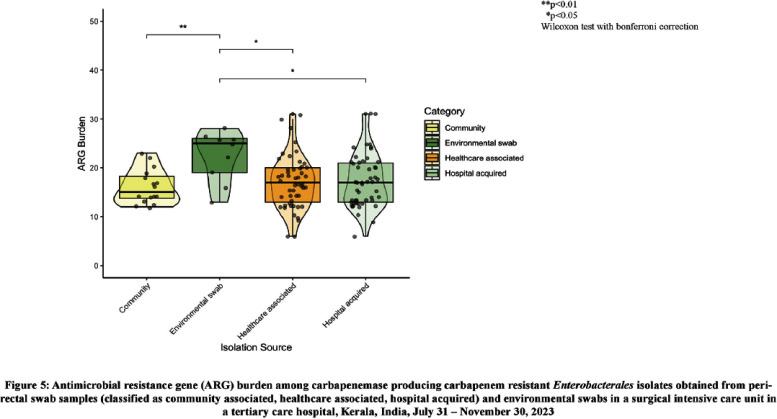

**Figure:**
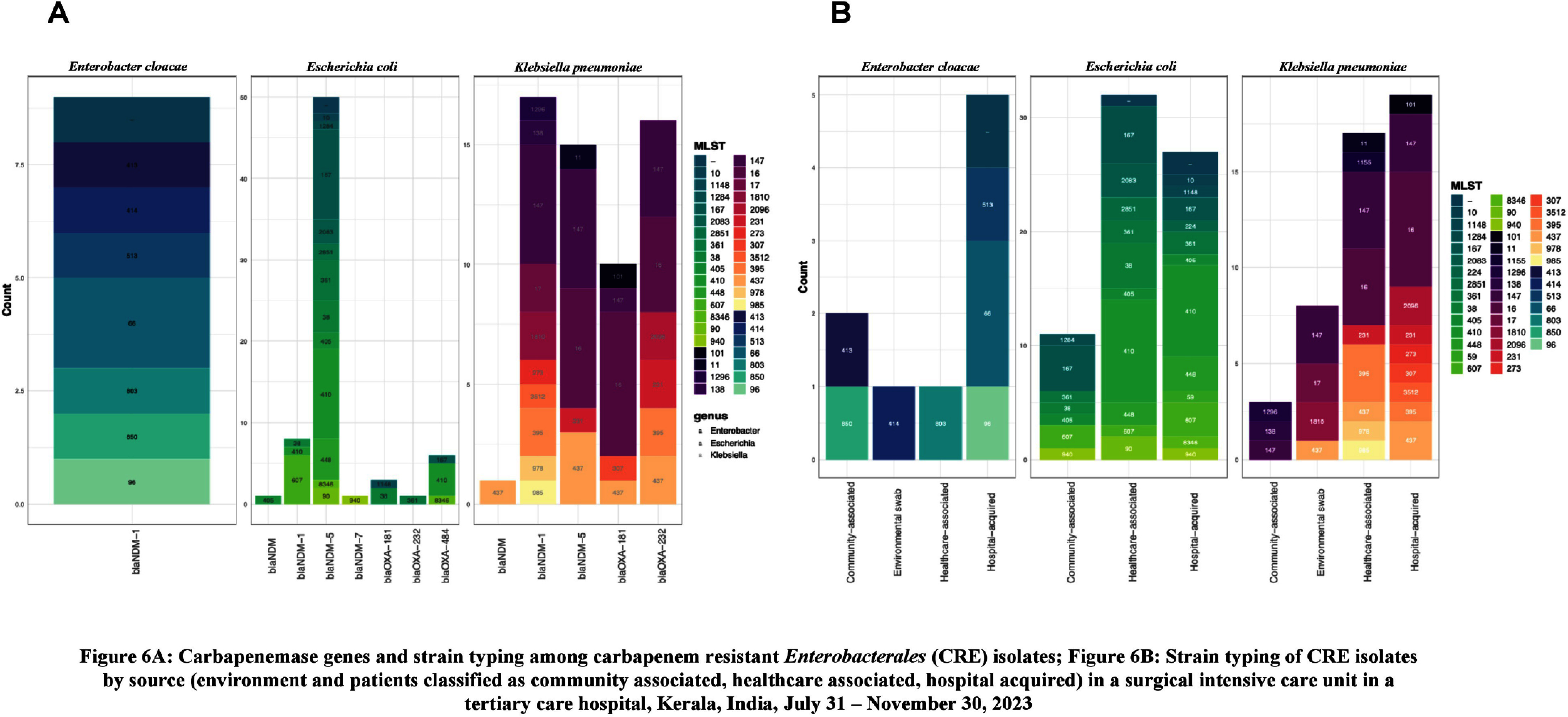

**Figure:**